# Video-assisted thoracoscopic surgery for adult benign idiopathic bronchoesophageal fistula: a report of two cases

**DOI:** 10.1093/icvts/ivaf114

**Published:** 2025-05-16

**Authors:** Qihang Zhu, Jing Zhan, Xiaojing Yao, Haiping Xiao

**Affiliations:** Department of Cardiothoracic Surgery, The First Affiliated Hospital of Guangdong Pharmaceutical University, Guangzhou, Guangdong, China; Department of Cardiothoracic Surgery, The First Affiliated Hospital of Guangdong Pharmaceutical University, Guangzhou, Guangdong, China; Department of Cardiothoracic Surgery, The First Affiliated Hospital of Guangdong Pharmaceutical University, Guangzhou, Guangdong, China; Department of Cardiothoracic Surgery, The First Affiliated Hospital of Guangdong Pharmaceutical University, Guangzhou, Guangdong, China

**Keywords:** bronchoesophageal fistula, idiopathic, video-assisted thoracoscopic surgery

## Abstract

We described two cases with idiopathic bronchoesophageal fistula presented recurrent postprandial coughing. Abnormal tracts connecting the oesophagus and bronchus were identified by videofluoroscopy. Thoracoscopic surgery was successfully performed, which involved the resection of the fistula and the interposition of a pedicle of viable parietal pleura between oesophageal and bronchial closures. Neither patient experienced symptoms of any subsequent complications.

## INTRODUCTION

Adult bronchoesophageal fistula (BEF) is a rare condition that is typically secondary to various pathologies, including tumours, trauma and diverticula [1, 2]. In certain patients diagnosed with idiopathic BEF based on symptoms and images, the exact aetiology remained undetermined despite a thorough evaluation. Traditionally, Surgical repair through thoracotomy has been the predominant surgical approach. With the advancement of thoracoscopic technology, numerous complex procedures can now be effectively performed using thoracoscopy. We reported two cases of idiopathic BEF repaired via thoracoscopic surgery.

## CASES REPORT

### Case 1

**Figure 1:**
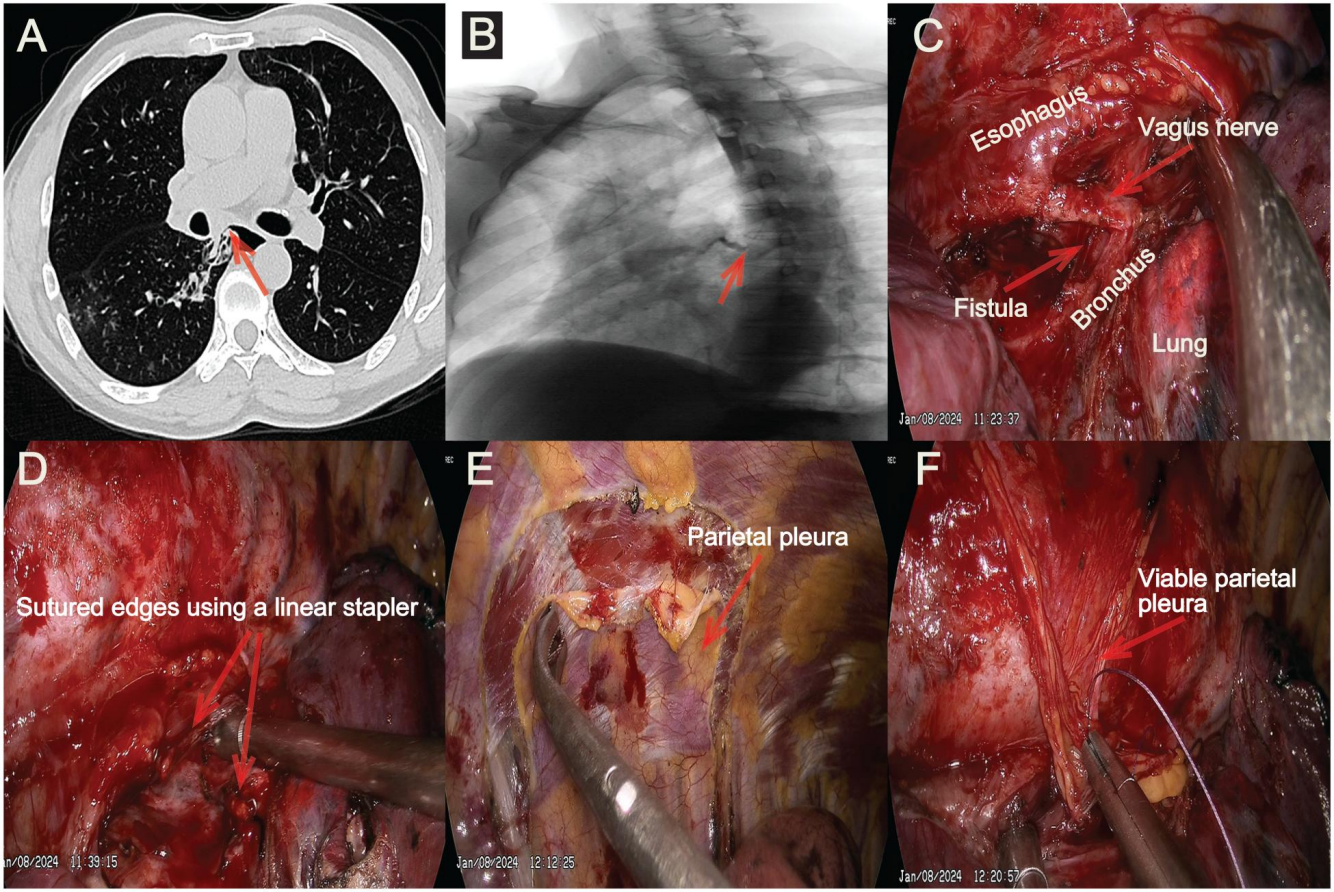
(**A**) Computed tomography revealed chronic right lower pneumonia and the abnormal tract (arrow). (**B**) Videofluoroscopy confirmed the fistula connecting the oesophagus and the right middle bronchus (arrow). (**C**) Identification and exposure of the anomalous fistula. (**D**) After being incised and sutured using a linear stapler. (**E**) Plan the pleural area based on the scope. (**F**) Using a piece of viable parietal pleura to interpose between oesophageal and bronchial closures

A 41-year-old man was admitted to our department on January 2nd, 2024 because of recurrent and worsening postprandial coughing for approximately 10 months. During this time, the patient underwent several treatments at local hospitals due to fever. However, each time was diagnosed with pneumonia based on chest X-ray. Computed tomography (CT) scan revealed chronic right lower pneumonia (Fig. 1A). Videofluoroscopy confirmed the abnormal tract connecting the oesophagus and the right middle bronchus（Fig. 1B）. Bronchoscopy indicated a 0.2 cm fistula proximal to the right middle bronchus. Gastroscopy revealed a 0.3 cm fistula located 28 cm from the upper incisor. Blood tests and physical examinations did not reveal any abnormal findings.

The surgery was performed on January 8th, 2024. The patient was positioned in the lateral decubitus position and underwent double-lumen endotracheal intubation under general anesthesia. A skin incision of approximately 30 mm was made at the junction of the midaxillary line and the fifth intercostal space. Posterior mediastinal pleura was dissected to isolate and expose the abnormal tract connecting the oesophagus and bronchus (Fig. 1C) according to the videofluoroscopy. Then, another 10 mm incision was made at the seventh intercostal space. Linear stapler (Ethicon) was inserted to cut the tract and suture the edges on both sides (Fig. 1D). After that, absorbable suture was used to reinforce both sides. A pedicle of viable parietal pleura was dissected and interposed between oesophageal and bronchial closures to reduce the probability of recurrence (Figure 1E and F). The operation lasted 175 minutes with blood loss of about 50 mL.

### Case 2

**Figure 2:**
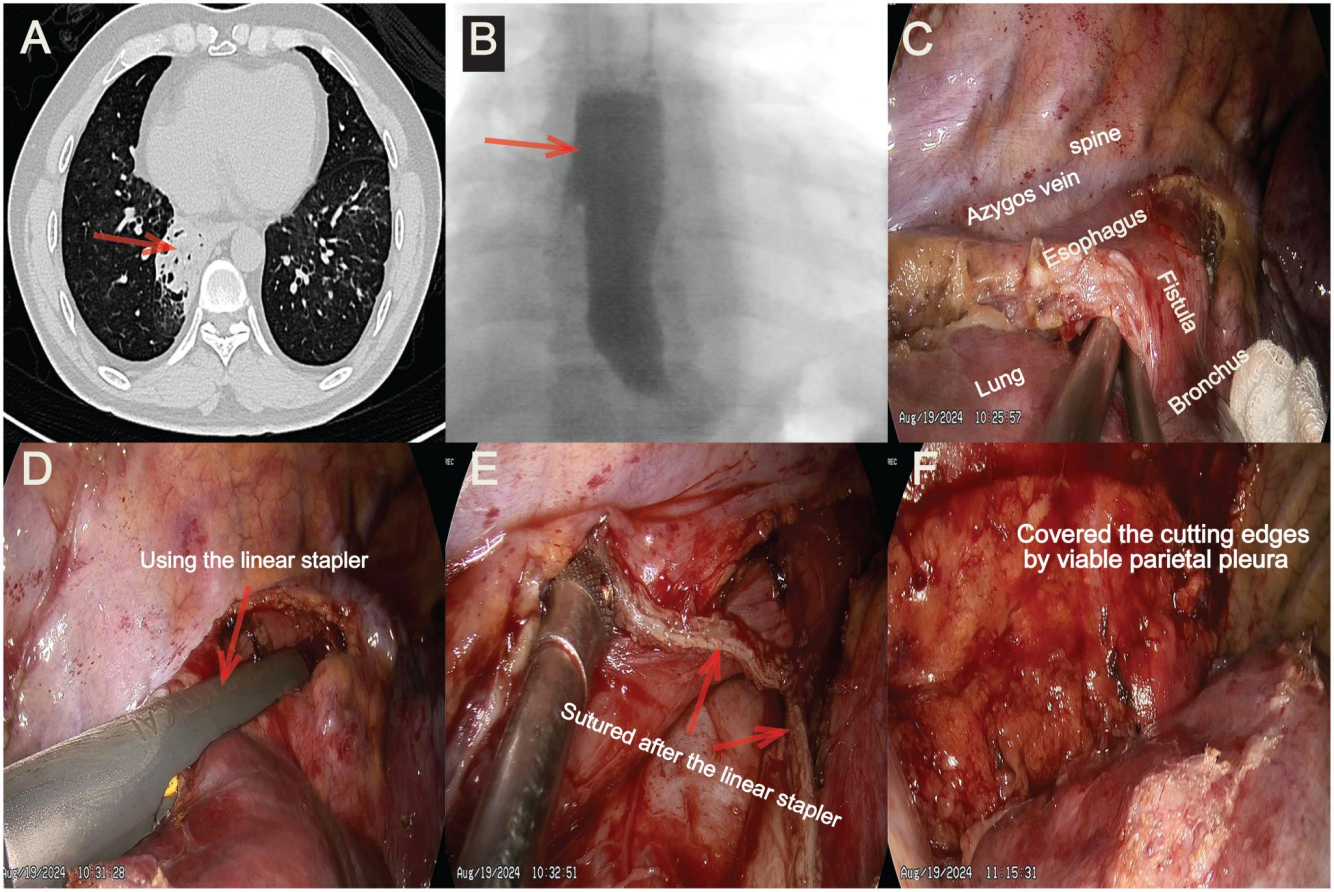
(**A**) Computed tomography revealed chronic right lower pneumonia (arrow). (**B**) Videofluoroscopy confirmed the abnormal tract connecting the oesophagus and the right middle bronchus (arrow). (**C**) Identification and exposure of the anomalous fistula. (**D**) Using a linear stapler to clip fistula. (**E**) After being incised and sutured. (**F**) After interpose the viable parietal pleura between oesophageal and bronchial closures

Another 52-year-old man was admitted with recurrent postprandial coughing for approximately 24 months on August 13th, 2024. CT revealed chronic right lower pneumonia, which is more consolidation than Case 1 (Figure 2A). Videofluoroscopy confirmed the fistula (Figure 2B). The invasive examinations, such as gastroscopy and bronchoscopy, were omitted. The same procedure lasted 160 minutes with blood loss of about 70 mL was performed (Figure 2C–F) on August 19th.

### Postoperative management

Both patients were fasted for 5 days postoperatively along with gastrointestinal decompression. Parenteral nutrition, acid inhibitors and short-term antibiotics were used during the period. No short-term complications occurred. Chest drainage tubes were removed on the postoperative third day. There were no abnormalities in the videofluoroscopy on 5th postoperative day. The gastric tubes were removed and the patients resumed a liquid diet. The patients were discharged on postoperative 7th day and instructed to gradually return to a normal diet. Up to January 2025, subsequent follow-up showed good life quality. No oesophageal stenosis or recurrence of fistula was observed.

## DISCUSSION

Neither patient had a history of endotracheal, oesophageal intubation, malignancy, infection or foreign bodies. The diagnosis and treatment of idiopathic BEF present considerable challenges. No standardized guidelines have been established due to the low incidence. Recurrent postprandial coughing and localized pneumonia at the same site indicate the possibility of idiopathic BEF. The most critical examination to confirm BEF is upper gastrointestinal videofluoroscopy. Gastroscopy and bronchoscopy would be beneficial, yet not essential [1].

There are very few cases concerning idiopathic BEF. Oesophageal diverticulum may be the primary potential cause of idiopathic BEF. Surgical repair is an effective approach for addressing BEF. The most widely adopted approach was thoracotomy, which often involved the interposition of vascularized muscle flaps [1, 2]. Thoracoscopic surgery has been widely used in thoracic diseases. Isolated cases have been reported and confirmed the use of thoracoscopic surgery in BEF [3, 4]. Muscle flaps are hard to obtain via thoracoscopy, whereas the pleura can be readily obtained. The viable parietal pleura, preserved by maintaining the pleural blood supply on the spinal side, serves as an appropriate tissue to be inserted between oesophageal and bronchial closures, thereby isolating tissues and reducing the risk of recurrence [5]. The whole operation process is minimally invasive and effective. Nevertheless, pre/intraoperative evaluation of the available tissue is important. Open surgery and muscle flap should be considered as alternative approaches.

There are limitations in our article. First, it is not feasible to conduct a comparative study, such as between open and thoracoscopic surgery, or between muscle and pleura, due to the limited number of cases. Second, follow-up time is limited. Long-term complications, including oesophageal stenosis, warrant continued observation. However, according to the current results, thoracoscopic surgery combined with pleural implantation demonstrates advantages and is worthy of further application and promotion in BEF.

## Data Availability

The data underlying this article will be shared on reasonable request to the corresponding author.
